# The Results of 16 Years of Iodization: Assessment of Iodine Deficiency Among School-age Children in Antalya, Turkey

**DOI:** 10.4274/jcrpe.galenos.2020.2019.0168

**Published:** 2020-09-02

**Authors:** Gamze Çelmeli, Yusuf Çürek, İkbal Özen Kücükçetin, Zümrüt Arslan Gülten, Sebahat Özdem, Sema Akçurin, İffet Bircan

**Affiliations:** 1 University of Health Sciences Turkey, Antalya Training and Research Hospital, Clinic of Pediatric Endocrinology, Antalya, Turkey; 2 Akdeniz University Faculty of Health Sciences, Department of Nutrition and Dietetics, Antalya, Turkey; 3 Akdeniz University Faculty of Medicine, Department of Pediatrics, Antalya, Turkey; 4 Akdeniz University Faculty of Medicine, Department of Biochemistry, Antalya, Turkey; 5 Akdeniz University Faculty of Medicine, Department of Pediatric Endocrinology, Antalya, Turkey

**Keywords:** Iodine deficiency, prevalence, school-age children, Turkey

## Abstract

**Objective:**

Iodine deficiency (ID) continues to be a problem around the world. This study investigated the prevalence of ID and goiter among school-age children in the city center of Antalya, Turkey. The aim was to investigate the effect of an iodization program, which had been running for sixteen years, on nutritional iodine status in this population.

**Methods:**

A total of 1,594 school children, aged 6-14 years, were included in this cross-sectional study. ID was evaluated based on median [interquartile range (IQR)] urine iodine/creatine (UI/Cr) (μg/g) ratio and median (IQR) UI concentrations (UIC) (μg/L). UICs were measured using the Sandell-Kolthoff method. Goiter was determined by palpation and staged according to World Health Organization classification.

**Results:**

Median (IQR) UIC was found to be 174.69 (119.17-242.83) μg/L, and UIC was found to be lower than 50 μg/L in 6.5% of the population. The median UI/Cr ratio increased from 62.3 to 163.3 μg/g and goiter rates had decreased from 34% to 0.3% over the 16 years of the program. However, 19% were still classified as ID (mild, moderate or severe) and, furthermore, 11.5% were classified as excessive iodine intake.

**Conclusion:**

Comparison of two cross-sectional studies, carried out 16-years apart, showed that Antalya is no longer an ID region. However, surveillance should be continued and the percentage of ID and iodine excess individuals in the population should be monitored to avoid emerging problems.

**What is already known on this topic?**

Despite highly effective salt iodization programs initiated by the World Health Organization, iodine deficiency continues to be a problem in some areas around the world.

**What this study adds?**

This study demonstrates that the 16-years iodization program allowed Antalya to become an iodine sufficient region with 174.69 (119.17-242.83) μg/L median (interquartile range) urinary iodine concentrations.

## Introduction

Iodine is an essential trace mineral required for the synthesis of thyroid hormones that play a critical role in normal growth and neurodevelopment in fetal life, infancy and childhood. Iodine deficiency (ID) causes a wide spectrum of pathologies throughout human development, from fetal life into adulthood. The clinical manifestations of ID disorders (IDD) include miscarriage, stillbirth, neurologic and myxomatous cretinism, goiter, hypothyroidism, mental retardation, intellectual impairment and impaired physical development (1,2).

Despite the significant increase in the number of iodine-sufficient countries as a result of the universal, largely effective salt iodization programs initiated by World Health Organization (WHO) and the International Council on Iodine Deficiency Disorders (ICCIDD) in 1990, in 2012 insufficient iodine intake was still reported in 29.8% (246 million) of school-age children (SAC) globally (3).

Knowledge of the prevalence and degree of ID is important, in that it provides information about the effectiveness of iodine prophylaxis in the population. The best indicators of iodine nutrition (IN) in a population are the percentage of households using adequately iodized salt, the prevalence of goiter and the median urinary iodine (UI) concentration (UIC) among SAC or pregnant women (4).

The table salt iodization program was launched officially in Turkey in 1998. Erdoğan et al (5) reported a 31.8% goiter rate and a 58% moderate to severe ID in the Turkish population prior to mandatory iodization. Eight years after mandatory iodization, Erdoğan et al (6) reported that 27.8% of the population continued to suffer from moderate to severe ID and ID remained a serious public health problem in Turkey. Erdoğan et al (7) have suggested that more than a decade of iodine prophylaxis would be needed to eradicate goiter in a moderately iodine-deficient region.

To the best of our knowledge, the only study of community-based ID and the prevalence of goiter in Antalya, Turkey was conducted by Semiz et al (8) in 1999, prior to the salt iodization program. Semiz et al (8) reported mild-to-moderate ID in Antalya, Turkey with a 34% prevalence of goiter, and a median UI/creatine (UI/Cr) ratio of 62.3 (10.7-136.9) µg/g among SAC.

The aim in the present study was to evaluate ID, as determined by UI levels, and to evaluate the prevalence of goiter through physical palpation, in the city center SAC population of Antalya. The results were then compared with those of the study conducted by Semiz et al (8). In this way, the IN status of the population 16 years after the launch of mandatory salt iodization was evaluated.

## Methods

A cross-sectional study was carried out between May and June 2015 and included 58 schools of the total 124 (46.8%) schools in the city center of Antalya, Turkey. For the study, 1,700 school children aged 6-14 years were selected from the total of 61,092 students attending the schools, using a probability proportional to population size (9) cluster sampling method. After excluding 108 participants with chronic disease, regular medication use or malnutrition, or those who could not provide urine samples under appropriate conditions, a total of 1,594 students were included in the study. None of the participants had been exposed to gadolinium, iodine or barium containing contrast material in the 96 hours prior to sample collection.

Written permission was taken from the Antalya Provincial Directorate of Health and the Antalya Province National Education Directorate. The study was approved by the Ethics Committee of Akdeniz University (decision no: 269 of 27.05.2015) and was supported by the Akdeniz University Scientific Research Projects Coordination Unit (project number: TSA-2016-962). Informed consent was obtained from the participants and their parents.

Early-morning spot urine samples were collected, ensuring no contamination, and goiter staging was assessed according to the WHO classification (grade 0: no goiter; grade 1: thyroid palpable but not visible; grade 2: thyroid visible with neck in normal position) by a pediatric endocrinologist. Urine samples were collected in deiodized test tubes, and were transferred immediately to the laboratory where they were stored at -20 °C.

The median UI/Cr (µg/g) and the median UIC (µg/L) were used in order to compare our results with the previous study conducted by Semiz et al (8) in Antalya and with other studies in literature, respectively. Severe ID was defined as median UIC <20 µg/L, moderate ID as 20-49 µg/L and mild ID as 50-99 µg/L. Optimal IN was defined as median UIC 100-199 µg/L, more IN than required as 200-299 µg/L and excessive IN as >300 µg/L (4).

Iodine levels in the urine were measured using the Sandell-Kolthoff method, as recommended by WHO and ICCIDD (4). Urine creatinine levels were measured spectrophotometrically using the Jaffe colorimetric method.

### Statistical Analysis

The data was analyzed using Statistical Package for Social Sciences software, version 22 (IBM Inc., Chicago, Ill., USA). The values in the text are presented as median, and interquartile range (25^th^ and 75^th^ percentiles; IQR), unless otherwise stated. The statistical analysis was performed using parametric (Student’s t-tests) or nonparametric (Mann-Whitney U tests) tests, when appropriate. Values of p<0.05 were accepted as statistically significant.

## Results

A total of 1,594 students, 839 male (52.6%) and 755 (47.4%) female, were included in the study. The mean±standard deviation age of the participants was 10.6±2.5 years.

The median (IQR) UIC was found to be 174.69 (119.17-242.83) µg/L among school children aged 6-14 years in the city center of Antalya, Turkey. The UIC was lower than 50 µg/L in 6.5% of the sample. The percentages of participants with mild, moderate, and severe ID and the IN status are shown in Table 1. Stage 1 goiter was detected in only five students (0.3%) upon physical examination.

When UI/Cr was evaluated for a comparison of the results with the 1999 study, the median (IQR) was found to be 163.3 (105.3-254.8) µg/g, and the UI/Cr ratio was found to be lower than 50 µg/g in 4.4% of the population using this method. The results of the comparison of the two cross-sectional studies carried out 16 years apart, that is before and after mandatory salt iodization, are presented in Table 2.

## Discussion

In the most recent cross-sectional study it was shown that 16 years after mandatory introduction of table salt iodization, Antalya has become an iodine-sufficient region, with a median (IQR) UIC of 174.69 (119.17-242.83 µg/L) among SAC. The UI/Cr ratio increased from 62.3 to 163.3 µg/g, the proportion of the population with below 50 µg/L UIC was found to be only 6.5% and the goiter rate decreased from 34% to 0.3% in 16 years (Table 1, 2). Accordingly, the WHO targets of median UIC between 100 µg/L and 299 µg/L among SAC, not more than 20% of UIC samples below 50 µg/L and total goiter rate below 5%, were met in the region (4).

Despite these positive results, as is the case in all iodine-sufficient countries, Turkey is suffering from some ongoing and newly emerging problems that must be taken into careful consideration. As more than 90% of dietary iodine is excreted in the urine, UIC is an useful biomarker of the current iodine intake of the population, and is the main indicator in assessments of the iodine status of a population (4,10). However, it is known that UIC can show high intra-individual variability, which may lead to reports of high percentages of individuals with inadequate IN in iodine-sufficient populations where most of the iodine intake comes from iodized salt (3). In our study, although the median UIC was 174.69 µg/L, 19% of the population was classified as ID (mild, moderate or severe) (Table 1). Prevalence assessments from 2012 estimate that 75% of the children who are affected by low iodine intake live in iodine sufficient countries (3,11). Gordon et al (12) found that iodine supplementation improves cognitive function, even in mildly iodine-deficient children, and so the iodine sufficient countries should also monitor the percentage of affected individuals.

The second problem is that, although the iodine status of SAC is generally representative of the adult population, this does not hold true for pregnant women and newborns. In a recent study, Erdoğan et al (7) reported a median UIC of 117 µg/L and goiter prevalence of 1.3% among SAC in Ankara, Turkey’s capital. Over a similar period, Oguz Kutlu and Kara (13) reported a median UIC of 80.5 µg/L and goiter prevalence of 15.4% among pregnant women in Ankara, and indicated that UIC was below 150 µg/L in 72.8% of pregnant women. In a recent large survey, the median UIC was found to be 73 µg/L among pregnant women in an iodine-sufficient metropolitan city. UIC was found to be below 50 µg/L in 36.6% of pregnant women and below 150 µg/L in 90.7% (14). There have been numerous studies to date highlighting the presence of ID in pregnant women and newborns in various countries where iodine appears to be adequate (15,16,17). These studies show that national iodization programs do not meet the increased iodine requirements of pregnant women. Accordingly, even iodine-sufficient countries should periodically screen these at-risk groups. In addition, there is a need for mandatory iodine supplementation programs for pregnant and lactating women.

Another population vulnerable to ID are those people living in rural areas. In the 2009 survey, Erdoğan et al (6) found a significant difference among rural and urban areas in Turkey. There are many studies highlighting the difference in prevalence of ID levels in rural areas (18,19). Therefore, it should be noted that this study does not reflect iodine status of rural populations in Antalya.

In societies where iodine intake is now sufficient, the fourth, newly emerging problem is excessive iodine intake (median UIC greater than 300 µg/L). Recent national surveys found excessive iodine intake in 10 countries around the world (4). This is reflected in the increase in number of studies investigating excessive iodine intake. Katagiri et al (20) showed in a meta-analysis that chronic exposure to excess iodine is a risk factor for hypothyroidism. The mechanism behind hypothyroidism is thought to involve an adaptation of the thyroid gland to excess iodine uptake (21,22). In China, the prevalence of subclinical hypothyroidism and thyroid nodules was found to be 20% and 15.5%, respectively, in areas with excess iodine intake, although the prevalence of subclinical and overt hyperthyroidism in the ID group was higher than in the excess iodine group (23). It has been shown that, after the start of mandatory salt iodization, the incidence of autoimmune thyroid disorder has increased in many countries (24,25). In the present study, UICs were 200-299 µg/L (more than required) in 28.1% of the population and were >300 µg/L (excessive IN) in 11.5%. Accordingly, surveillance studies should be continued in iodine sufficient regions to prevent the growth of excess iodine as a problem.

Measuring the ratio of UI to creatinine for population screening is expensive, and can be misleading in some cases, such as in the presence of malnutrition, and so is no longer recommended (4,26). In the present study, however, in order to be comparable with a previous study conducted in Antalya, we evaluated both UI/Cr ratios as well as UICs.

### Study Limitations

In our study, we detected goiter in 0.3% of the population upon physical examination. Although palpation is the traditional diagnostic method, the sensitivity and specificity of palpation are poor in mild to moderate ID areas. Percentages may be higher when thyroid size is measured by ultrasound. The strength of this study was its detailed demonstration of the outcome of 16 years of mandatory salt iodization in the same geographical region.

## Conclusion

Our study shows that as a result of the application of an effective salt iodization program 16 years previously, Antalya is now an iodine sufficient region. Surveillance studies should be continued in SAC and in at-risk groups to ensure adequate iodine intake. The levels of ID and excess iodine intake in the population should be carefully monitored to avoid newly emerging problems.

## Figures and Tables

**Table 1:**
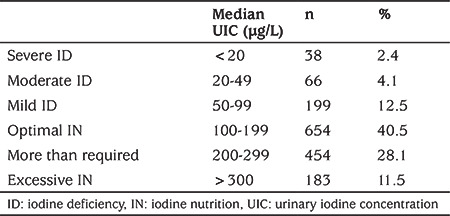
The distribution of iodine deficiency and iodine nutrition status in the city center of Antalya, Turkey

**Table 2:**
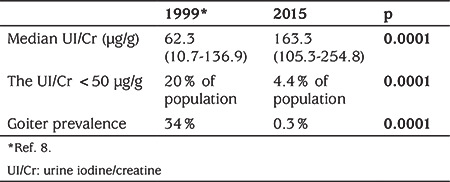
Comparison of two cross-sectional studies, carried out one year and 16-years after introduction of a national salt iodization programme, in Antalya, Turkey
